# Association of *CYP2C19* Polymorphisms with the Clinical Efficacy of Clopidogrel Therapy in Patients Undergoing Carotid Artery Stenting in Asia

**DOI:** 10.1038/srep25478

**Published:** 2016-05-03

**Authors:** Wen-Yao Zhu, Ting Zhao, Xiao-Yi Xiong, Jie Li, Li Wang, Yu Zhou, Zi-Li Gong, Sai-Yu Cheng, Yong Liu, Jie Shuai, Qing-Wu Yang

**Affiliations:** 1 Department of Neurology, Xinqiao Hospital, the Third Military Medical University, Chongqing, 400037, China

## Abstract

The *CYP2C19* gene plays a detrimental role in the metabolism of clopidogrel. This study aimed to investigate the association between *CYP2C19* polymorphisms and the clinical efficacy of clopidogrel therapy in patients who have undergone carotid artery stenting (CAS). *CYP2C19* genotype screening was performed on 959 ischemic stroke patients. Of these patients, 241 who had undergone CAS were enrolled in the study. They were all followed up for 1 year after stent surgery, and the primary clinical end-points were ischemic events. The frequencies of the CYP2C19*2 and *3 alleles among the 959 patients were 31.80% and 5.06%, respectively. Regarding the 241 participants who had undergone CAS, multivariate Cox regression analysis showed that the *CYP2C19* loss-of-function (LOF) alleles (*2 and *3) were risk factors for post-CAS prognosis. Within 1 year of follow-up, the patients carrying the *CYP2C19* LOF alleles were more likely to experience ischemic events than those carrying none. The occurrence of ischemic events did not significantly differ between the *2 and *3 allele carriers. Our results suggest that *CYP2C19* LOF alleles (*2 and *3) significantly impact the prognosis of patients on clopidogrel therapy after CAS and that the CYP2C19*2 and CYP2C19*3 alleles have the same effects on prognosis.

Carotid artery atherosclerotic stenosis is an important risk factor for ischemic stroke. Approximately 20% of ischemic stroke cases are due to focal atherosclerosis and consecutive narrowing (stenosis) of the internal carotid artery^1^,^2^,^3^. Carotid artery stenting (CAS) is an alternative option for treating carotid atherosclerotic stenosis^4^,^5^,^6^,^7^. Although CAS is associated with increases in the minor stroke rate and cost, it possesses the advantages of noninvasiveness and easy recovery. After CAS, patients must be administered a long-term regimen of clopidogrel for antiplatelet therapy^8^,^9^,^10^. Clopidogrel is a prodrug that requires hepatic cytochrome P450 (CYP) for its conversion into an active metabolite^11^. *CYP2C19* is one of the principal CYP enzymes that affect the metabolism of clopidogrel. At least 25 genetic variants of *CYP2C19* have been identified^12^. Among them, the wild-type CYP2C19*1 allele is associated with functional *CYP2C19*-mediated metabolism, while the most common *CYP2C19* loss-of-function (LOF) variant alleles are CYP2C19*2 and *3, which account for the majority of defective genotypes^13^. The *CYP2C19* LOF alleles (*2 and *3) reduce the level of the active metabolite of clopidogrel in the blood, which contributes to a decrease in the function of clopidogrel, resulting in inhibiting platelet aggregation^14^,^15^,^16^. Furthermore, the allelic frequencies of *CYP2C19* variants display significant interethnic differences. Compared to other racial groups, the frequencies of the CYP2C19*2 (681 G > A change in exon 5)^17^ and CYP2C19*3 (636 G > A change in exon 4)^18^ alleles in Asian populations are significantly elevated^19^,^20^. This indicates that individuals of Asian descent are more likely to be resistant to clopidogrel.

Several large cohort studies have reported that patients with the CYP2C19*2 or CYP2C19*3 allele who have undergone percutaneous coronary intervention (PCI) are more likely to experience worse clinical outcomes during clopidogrel therapy^15^,^21^,^22^,^23^. A study of acute ischemic stroke has also shown that patients with *CYP2C19* LOF alleles have a reduced response to clopidogrel and experience a significantly poorer outcome, according to modified Rankin Scale (mRS) scores, at 3 and 6 months after stroke^24^. However, it is not yet clear whether the *CYP2C19* LOF alleles (*2 and *3) have an impact on the clinical efficacy of clopidogrel therapy after CAS. In this study, we selected patients with *CYP2C19* LOF alleles who had undergone CAS for enrollment to determine the relationship between *CYP2C19* gene polymorphisms and the clinical efficacy of clopidogrel therapy after CAS. The findings of this study could provide guidance for the development of clinical personalized antiplatelet therapy after CAS.

## Results

### *CYP2C19* genotype frequencies

From November 2012 to June 2014, 959 ischemic stroke patients underwent *CYP2C19* genotype screening at our hospital. We found that 61.21% of the patients carried at least one *CYP2C19* LOF allele, including 12.52% patients carrying 2 LOF alleles. The *CYP2C19* *1/*2 genotype was the most predominant among the patients (42.44%), followed by the *CYP2C19* *1/*1 genotype (38.79%). We also calculated CYP2C19*2 allele frequencies by genotype (including *1/*2, *2/*2 and *2/*3), and we performed the same calculation for CYP2C19*3 allele frequencies by genotype (including *1/*3, *2/*3 and *3/*3). The frequency of the CYP2C19*2 allele was 31.80%, and that of the CYP2C19*3 allele was 5.06%. Different phenotypes were predicted by the number of LOF alleles. Patients with 2 LOF alleles were poor metabolizers (PM), patients with 1 LOF alleles were intermediate metabolizers (IM), and non-carriers were extensive metabolizers (EM)^24^ (Table 1).

### Baseline characteristics

According to the differences in their *CYP2C19* genotypes, the 241 patients undergoing CAS were divided into the following 5 groups (none of the patients had the *3/*3 genotype) : *1/*1 (n = 89), *1/*2 (n = 116), *1/*3 (n = 16), *2/*2 (n = 16), and *2/*3 (n = 4). The average patient age was 64.3 years (SD, 9.3), 90% of the patients were men, and the percentage of patients with a degree of stenosis of over 70% was 62.7%. Almost all of the patients received dual antiplatelet therapy during the pre-operative period. No differences were observed in the baseline characteristics among the 5 groups (Table 2).

### Risk factors for the primary end points

The multivariate Cox regression model was used to determine the independent risk factors for the primary end-points. We included age, sex, smoking, hypertension, hyperlipidemia, and diabetes in multivariate Cox regression analysis to identify independent correlates of the primary end-points (Table 3). The results showed that the *CYP2C19* LOF alleles (*2 and *3) were risk factors for post-CAS prognosis (relative risk, 2.411; 95% CI, 1.050 to 5.537; P = 0.038).

### Clinical end-points

**Figure 1:**
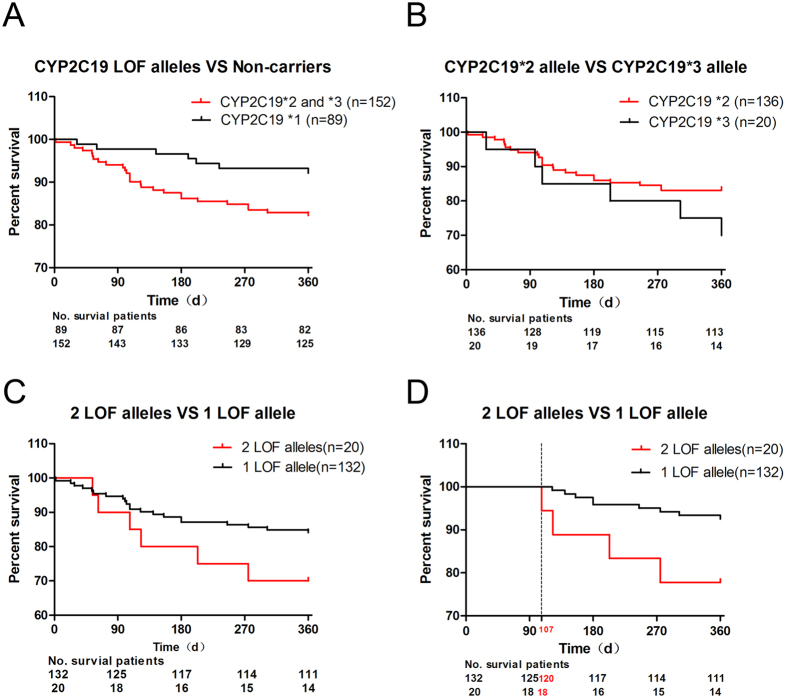
Rates of recurrence of ischemic event-free survival over 1 year of follow-up. (**A**) *CYP2C19* LOF alleles including the genotypes *1/*2, *1/*3, *2/*2 and *2/*3. The genotype of non-carriers is *CYP2C19* *1/*1. The curves represent the percentage of patients surviving at the endpoints. The numbers below the survival curves are the numbers of patients in each group who survived at the endpoints and were still at risk over the follow-up period. (**B**) The cumulative survival rates did not differ between the patients with the CYP2C19*2 and CYP2C19*3 alleles. These alleles are both included in the genotype *2/*3; therefore, patients with this genotype are counted twice. (**C,D)** The rates of recurrence of ischemic event-free survival did not differ between the two groups during the first 107 days post-CAS. After postoperative day 107, these rates were significantly different between the groups.

Based on the results obtained using the Cox regression model, we explored the respective effects of the different *CYP2C19* genotypes on the clinical prognosis of the patients treated with clopidogrel after CAS. Over the 1 year follow-up period, the primary endpoint of ischemic events occurred in 7 (7/89) patients with the *CYP2C19* *1/*1 genotype, 21 carrying 1 *CYP2C19* LOF allele (17 patients with *1/*2 and 4 with *1/*3), and 6 carrying 2 *CYP2C19* LOF alleles (4 patients with *2/*2 and 2 with *2/*3). First, we divided the 241 patients who had undergone CAS into the following two groups according to whether they carried *CYP2C19* LOF alleles: a group of patients with *CYP2C19* LOF alleles (*2 and *3) and a group of non-carriers (*1/*1). We generated Kaplan-Meier curves to compare the 1-year recurrence of ischemic event-free survival. The curves showed that the patients carrying *CYP2C19* LOF alleles (*2 and *3) were more likely to experience ischemic events than the non-carriers (hazard ratio, 2.131; 95% CI of the ratio, 1.067 to 4.255; P = 0.032; Fig. 1A). Second, we evaluated whether the CYP2C19*2 and CYP2C19*3 alleles had the same effect on the primary end-points. The results showed that the influences of the CYP2C19*2 and CYP2C19*3 alleles on clinical events did not differ (hazard ratio, 0.4708; 95% CI of the ratio, 0.1562 to 1.419; P = 0.1808; Fig. 1B). Finally, we divided the patients into two groups according to the number of carried LOF alleles as follows: 1 *CYP2C19* LOF allele (n = 132) and 2 *CYP2C19* LOF alleles (n = 20). No differences were observed in the clinical end-points between the two groups (hazard ratio, 2.441; 95% CI of the ratio, 0.7786 to 7.651; P = 0.1258; Fig. 1C). However, according to the trends observed in the survival curves, at almost 100 days, the cumulative survival rates significantly differed among the groups. Therefore, we performed further analysis and found that after postoperative day 107, the risk of cumulative ischemic events showed an increasing trend according to the number of *CYP2C19* LOF alleles (hazard ratio, 6.176; 95% CI of ratio, 1.145 to 33.32; P = 0.0342; Fig. 1D).

## Discussion

In our study, we first confirmed the association between *CYP2C19* polymorphisms and the clinical efficacy of clopidogrel therapy in patients who had undergone CAS in China. A total of 241 patients who had undergone CAS were enrolled in this study and were divided into 5 groups according to their *CYP2C19* genotypes. The multivariate Cox regression model showed that the *CYP2C19* LOF alleles (*2 and *3) were risk factors for post-CAS prognosis. The results of Kaplan-Meier curve analysis of the clinical end-points also confirmed this finding by revealing that the patients carrying the *CYP2C19* LOF alleles (*2 and *3) were more likely to experience ischemic events than the non-carriers. These results all demonstrate that the *CYP2C19* LOF alleles (*2 and *3) are risk factors for the prognosis of patients who have undergone CAS and are on clopidogrel therapy.

In our study, the allelic frequency of CYP2C19*2 was 31.80% and that of CYP2C19*3 was 5.06%. Previous studies have shown that the allelic frequency of CYP2C19*2 in Asian populations is approximately 30%, and that of CYP2C19*3 is approximately 10%^19^,^20^. Furthermore, a study of an East Asian population^16^ has reported that the frequencies of the CYP2C19*2 and CYP2C19*3 alleles are 28.6% and 8.3%, respectively. The CYP2C19*2 allelic frequency observed in our study is in line with these previous findings; however, the CYP2C19*3 allelic frequency is lower than previously reported. The study of the East Asian population^16^ enrolled 266 Korean patients, whereas we enrolled 959 Chinese patients for *CYP2C19* gene screening. Therefore, the CYP2C19*3 allelic frequency may have been lower in our study than in the previous study because of sample size and regional differences. The allelic frequencies of CYP2C19*2 in Caucasian and African-American populations are 13% and 18%^19^,^20^, respectively. In addition, those of CYP2C19*3 in these populations are less than 1%^19^,^20^. Compared to other racial groups, the *CYP2C19* LOF alleles occur more frequently in Asian populations.

With regard to the primary clinical end-points, we found that the influences of the CYP2C19*2 and CYP2C19*3 alleles on clinical events did not differ, which is in agreement with the findings of Jeong, Y. H. *et al.*^16^, who also noted that the occurrence of cardiovascular events increased according to the number of *CYP2C19* LOF alleles. However, in our study, the number of *CYP2C19* LOF alleles only had an effect on the end-points after day 107. There are two possible reasons for the lack of statistical significance observed during the first 107 days post-CAS. The first reason may be that a limited sample size was studied, and the second may be that aspirin was used for first 90 days after CAS. Thus a large sample study is required for further validation of these findings.

With regard to the secondary clinical end-points, two patients experienced cerebral hemorrhage during the follow-up period, and the genotypes of these patients were *1/*1 and *1/*2. Because of the relatively small sample size, we are not sure whether these *CYP2C19* gene polymorphisms are associated with hemorrhagic stroke. We also determined the National Institutes of Health Stroke Scale (NIHSS) and mRS scores of the patients who had undergone CAS over the 1 year of follow-up. As the ischemic symptoms of the patients who had undergone CAS had improved greatly during the post-operative period, the scores among the different groups divided according to the *CYP2C19* genotype did not statistically significantly differ.

This was a single-center study, so it has some limitations. First, all of the patients were enrolled from our hospital. However, China is a multi-ethnic country. Therefore, the population enrolled and the frequencies of *CYP2C19* genotypes observed in the current study, are the most representative of the Han nationality in southwest China. Thus, there might have been selection bias in the choice of the population. Further multi-center, large sample studies are needed to expand upon our findings. In addition, a previous study has reported that *CYP2C19* genotypes can affect the pharmacokinetics and pharmacodynamics of clopidogrel, for example, by reducing the level of the active metabolite of clopidogrel^14^,^15^ and by markedly influencing ADP-induced platelet aggregation^21^,^25^. However, part of this study included retrospective analysis, and we were not able to obtain complete relevant data from our stroke database to confirm these previous findings. Furthermore, patients enrolled in some studies were given a 300–600 mg oral loading dose of clopidogrel followed by 75 mg per day before the PCI^15^,^21^. And platelet reactivity testing is not recommended before day five unless using a starting dose of 300 or 600 mg at day 1 due to the feared “false rate of non- or low- responders”. These phenomena might were caused by that *CYP2C19* LOF alleles are associated with a marked decreased in ADP-induced platelet aggregation^21^,^25^. As we have not do the platelet reactivity test, we could only according to the guidelines^10^ gave the patients with 75 mg clopidogrel daily for 3–5 days before the intervention.

In conclusion, we found that patients who have undergone CAS and carry *CYP2C19* LOF alleles (*2 and *3) are more likely to experience ischemic events during clopidogrel therapy. For these patients, a high dose of clopidogrel should not be used. Future studies are recommended to valuate alternative antiplatelet drugs, such as ticagrelor, prasugrel or additional cliostazol^26^. In addition, patients should be watched for bleeding events, because ticagrelor and prasugrel are associated with increased bleeding rate^26^.

## Methods

### Study Populations

For *CYP2C19* gene screening, between November 2012 and June 2014, 959 ischemic stroke patients at our hospital were found to meet the following inclusion criteria: 1) a clinical diagnosis of ischemic stroke^27^; and 2) long-term use of clopidogrel as the main drug for ischemic stroke prevention. The exclusion criterion was cerebral embolism.

**Figure 2:**
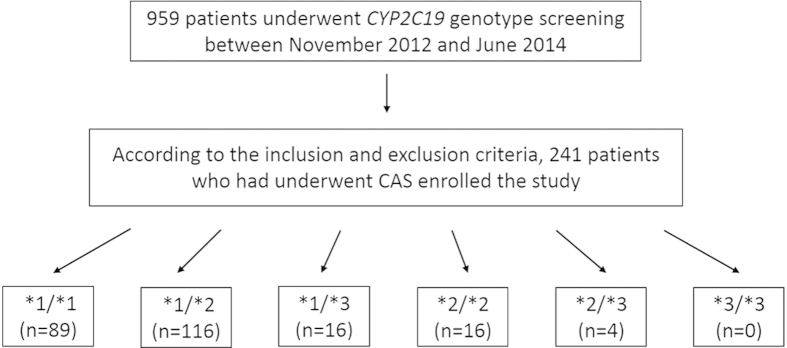
Flow diagram of the study population recruitment process. CAS indicates carotid artery stenting. *1/*1, *1/*2, *1/*3, *2/*2, *2/*3 and *3/*3 are the different *CYP2C19* genotypes.

Among the 959 patients who underwent *CYP2C19* genotype screening, a total of 241 were selected for enrollment in the study according to the inclusion and exclusion criteria (Fig. 2). The inclusion criteria were as follows: 1) carotid artery atherosclerotic cerebrovascular disease requiring CAS (with a reduction in the diameter of the lumen of the internal carotid artery of more than 70%, as documented by noninvasive imaging, or by more than 50%, as documented by catheter angiography, and an anticipated rate of periprocedural stroke or mortality of less than 6%^10^); 2) an age of 18–80 years old; 3) the receipt of clopidogrel for long-term antiplatelet therapy after post-procedural dual antiplatelet therapy; and 4) the receipt of *CYP2C19* gene screening. The exclusion criteria were as follows: 1) contraindication to antiplatelet therapy; 2) requirement for a carotid stenting because of carotid artery dissection; and 3) the use of additional antiplatelet drugs or anticoagulant drugs post-CAS. The 241 patients who had undergone CAS were divided into 6 groups according to the different *CYP2C19* genotypes.

This study was approved by the Medical Ethics Committee of Xinqiao Hospital, Third Military Medical University. Informed consent was obtained from all participants or their authorized family members. The study protocol was performed in accordance with relevant ethical guidelines and regulations for human studies.

### Dosage and Administration

Dual antiplatelet therapy consisting of 75 mg clopidogrel and 100 mg aspirin daily, beginning at 3–5 days before the intervention, was administered to all patients in this cohort. Dual antiplatelet therapy was also used after CAS, with a recommended duration of 3 months or at least 1 month^10^. After completing the dual antiplatelet therapy, all patients received 75 mg clopidogrel daily for long-term antiplatelet therapy^8^,^9^,^10^.

### Genetic Analysis

Genotyping (CYP2C19*1, CYP2C19*2, and CYP2C19*3) was performed using a commercially available kit (BaiO Technology Co, Ltd, Shanghai, China) after the extraction of genomic DNA from whole blood^16^,^24^, collected from the patients enrolled in the study and kept an anticoagulant tubes. We used a nucleic acid extracting reagent (BaiO Technology Co, Ltd, Shanghai, China) to obtain high-purity genomic DNA from the whole-blood samples. Polymerase chain reaction (PCR) was performed according to the following protocol: 50 °C for 5 minutes, 94 °C for 5 minutes, and 35 cycles at 94 °C for 25 seconds, 48 °C for 40 seconds, and 72 °C for 30 seconds, followed by elongation at 72 °C for 5 minutes. We obtained images of the hybridization of the amplification products with the gene probes. The images and data were analyzed using BaiO Array Doctor Version2.0 (BaiO Technology Co, Ltd, Shanghai, China) software and BSE 040101 (BaiO Technology Co, Ltd, Shanghai, China) software.

### End-points and Follow-up

The primary clinical end-points were ischemic events, including 1) previous ischemic symptom recurrence, 2) previous cerebrovascular transient ischemic attack (TIA), 3) stent thrombosis, 4) new cerebral infarction caused by previous cerebrovascular, and 5) death. The secondary clinical end-point was hemorrhagic stroke.

We recorded whether the patients experienced a clinical end-point within 1 year after CAS. The patients were followed-up at the 1st, 3^rd^ and 6th months, and at 1 year after CAS, because they were required to return to the hospital for regular check-ups during this time period. Additional information was obtained via telephone or from outpatient medical records as necessary.

### Statistical Analysis

All data were analyzed using SPSS version 19.0 software. Measurement data are presented as the mean ± SD. Furthermore, the data were compared using one-way ANOVA test. Enumeration data are presented as numbers or percentages, and were compared using the chi-square test or Fisher’s exact test. Multivariate Cox regression analysis of independent risk factors was performed to assess the primary end-points. Kaplan-Meier curves were generated to compare the 1-year recurrence of ischemic event-free survival among the patients with the different *CYP2C19* genotypes. A P value of <0.05 was considered statistically significant.

## Additional Information

**How to cite this article**: Zhu, W.-Y. *et al.* Association of *CYP2C19* Polymorphisms with the Clinical Efficacy of Clopidogrel Therapy in Patients Undergoing Carotid Artery Stenting in Asia. *Sci. Rep.* **6**, 25478; doi: 10.1038/srep25478 (2016).

## Figures and Tables

**Table 1:** Genotypic and allelic frequencies and phenotypes of ischemic stroke patients.

Gene	Genotype	Distribution, n (%)	Predicted Phenotype	Allele	Frequency, %
*CYP2C19*	*1/*1	372 (38.79)	extensive metabolizer	*1	63.14
	*1/*2	407 (42.44)	intermediate metabolizer	*2	31.8
	*1/*3	60 (6.25)	intermediate metabolizer	*3	5.06
	*2/*2	88 (9.18)	poor metabolizer		
	*2/*3	27 (2.82)	poor metabolizer		
	*3/*3	5 (0.52)	poor metabolizer		

**Table 2:** Baseline characteristics of patients who had undergone CAS.

Variables	Total (n = 241)	*1/*1 (n = 89)	*1/*2 (n = 116)	*1/*3 (n = 16)	*2/*2 (n = 16)	*2/*3 (n = 4)	p
Age, y	64.3 ± 9.3	64.6 ± 9.0	64.0 ± 9.8	67.6 ± 9.5	62.3 ± 7.3	62.0 ± 5.4	0.506
Male, n (%)	217 (90.0)	82 (91.3)	110 (94.8)	13 (81.2)	13 (81.2)	4 (100)	0.151
Degree of stenosis (%)							
50–70%	90 (37.3)	32 (36.0)	45 (38.8)	4 (25.0)	8 (50.0)	1 (25.0)	0.631
>70%	151 (62.7)	57 (64.0)	71 (61.2)	12 (75.0)	8 (50.0)	3 (75.0)	0.631
Risk factor, n (%)							
Diabetes mellitus	54 (22.4)	18 (20.2)	27 (23.3)	3 (18.75)	3 (18.75)	3 (75.0)	0.141
Hypertension	155 (64.3)	59 (66.3)	70 (60.3)	12 (75.0)	12 (75.0)	2 (50.0)	0.575
Hyperlipidemia	99 (41.1)	31 (34.8)	48 (41.4)	7 (43.8)	11 (68.8)	2 (50.0)	0.154
Current smoking	120 (49.8)	45 (50.6)	61 (52.6)	7 (43.8)	6 (37.5)	1 (25.0)	0.633
Preoperative medicine, n (%)							
Clopidogrel	238 (98.8)	88 (98.9)	115 (99.1)	15 (93.8)	16 (100)	4 (100)	0.454
Aspirin	228 (94.6)	82 (91.3)	110 (94.8)	16 (100)	16 (100)	4 (100)	0.537
Statin	233 (97.7)	86 (96.7)	113 (97.4)	15 (93.8)	16 (100)	3 (75.0)	0.134
WBC count, ×10^3^/mm^3^	7.0 ± 2.2	6.8 ± 2.0	7.2 ± 2.2	6.7 ± 2.3	6.8 ± 2.6	5.4 ± 1.4	0.381
Hemoglobin, g/L	134.2 ± 16.3	134.5 ± 17.3	135.2 ± 16.6	132.9 ± 11.8	128.2 ± 14.4	127.3 ± 1.3	0.478
Platelet count, ×10^3^/mm^3^	183.2 ± 55.9	182.6 ± 60.5	185.6 ± 56.1	176.9 ± 37.7	175.3 ± 51.4	185.8 ± 19.4	0.971
Total cholesterol, mmol/L	4.1 ± 1.1	4.2 ± 1.2	4.4 ± 1.1	4.5 ± 1.2	5.1 ± 0.9	4.5 ± 1.5	0.061

The values are the mean ± SD or n (%). CYP2C19*3/*3 (n = 0).

**Table 3:** Risk factors for primary end point events.

Variable	Relative risk	95% CI	P
Age	0.982	0.944–1.021	0.364
Male	1.104	0.419–2.907	0.842
Smoking	0.714	0.319–1.598	0.412
Drinking	0.771	0.327–1.814	0.551
Family history	0.704	0.165–3.009	0.636
Hypertension	1.053	0.510–2.176	0.889
Hyperlipidemia	1.457	0.727–2.919	0.288
Diabetes mellitus	1.165	0.532–2.551	0.703
*CYP2C19* LOF alleles	2.411	1.050–5.537	0.038
